# A Three-Stage Model for the Acquisition of Anticipatory Planning Skills for Grip Selection during Object Manipulation in Young Children

**DOI:** 10.3389/fpsyg.2016.00958

**Published:** 2016-07-05

**Authors:** Kathrin Wunsch, Matthias Weigelt

**Affiliations:** ^1^Sportpsychology, Institute of Sport and Sport Science, University of FreiburgFreiburg, Germany; ^2^Sportpsychology, Department Sport and Health, University of PaderbornPaderborn, Germany

**Keywords:** end-state comfort effect, motor planning, motor development, anticipatory planning, degrees of freedom

When people manipulate objects, they plan their movements in advance of the execution in order to reach a desired goal or goal state at the end of the action. This can be nicely illustrated by the end-state comfort effect (ESC; Rosenbaum et al., 1990). For example, when people reach for an inverted cup, they will anticipate the final part of the manual rotation and are willing to adopt an initial awkward thumb-down grasp to end the rotation maneuver in a comfortable thumb-up posture (overturned-glass-task, OGT; Fischman, 1997). To this end, the grasping action is planned by selecting a particular final posture out of a set of stored postures (Rosenbaum et al., 2001). According to the “concept of order of planning” by Rosenbaum et al. (2012), such an anticipatory strategy is reminiscent of second-order planning, which entails not only planning for immediate task demands (as in first-order planning), but also considers what one wants to do with the object afterwards, such as holding the cup comfortable to pour tea into it.

There is a large body of research documenting that the ESC effect is present in adults within a number of different motor tasks (see Rosenbaum et al., 2012, for a review), and even in different non-human primates (e.g., Weiss et al., 2007; Chapman et al., 2010). Therefore, it is somewhat surprising that young children do not show the ESC effect reliably (Weigelt and Schack, 2010; Jovanovic and Schwarzer, 2011). In fact, studies have identified a rather protracted developmental trajectory, with children reaching adult levels around the age of 10 and older (Thibaut and Toussaint, 2010; Stöckel et al., 2012; Scharoun and Bryden, 2014). While today there is much evidence on the development of the ESC effect in young children (for a review see Wunsch et al., 2013), so far, there is no theoretical model on how these motor planning skills are acquired. In the present paper, we attempt to provide a model to further the discussion on the development of the ESC effect. To this end, we outline a three-stage developmental model on the acquisition of anticipatory planning skills for grip selection in object manipulation (as signified by the ESC effect) across childhood.

The general framing of the model and the separate skill acquisition stages in young children are primarily based on two different concepts to approach the control of complex human behavior: The *Degrees of Freedom* (DOF) problem (also referred to as the motor equivalence problem) has been introduced by Bernstein (1967). This concept addresses motor control in two ways: First, it can be used to describe the restriction of movements by limiting the range of motion for individual joints whenever people perform novel motor actions. This “freezing” of individual DOF results in lower movement variability and in better task control during early learning stages. As the learning process continues, more DOF will be exploited, which often results in a reorganization of motor behavior and in new task solutions. Second, it accounts for the strategy to limit the variety of motor actions used to accomplish a certain task. For example, limiting the infinite posture space to two postures (i.e., thumb-up and thumb-down grasp) reduces planning costs and supports the automatic grip selection as the behavioral control becomes more efficient and movement execution more skillful. As we will argue below, both strategies are employed during the acquisition of anticipatory planning skills for grip selection in young children. The *Anticipatory Behavioral Control* (ABC) framework has been proposed by Hoffmann and colleagues (Hoffmann, 2003; Hoffmann et al., 2007). It focuses on two learning mechanisms, which provide the cognitive structure for the anticipatory control of goal-directed actions. First, action-effect associations are acquired during early motor learning based on the contingency with which a particular motor action will produce a certain action effect (in the environment). Second, these action-effect associations are contextualized to specific situational conditions defining the behavioral context, which systematically modulate the contingencies between actions and effects as the learning process continues. Thus, the ABC theory takes “the primacy of action-effect learning as well as the conditionalization of action-effect relations” (Hoffmann et al., 2007, p. 134) into account, enabling the effortless selection of a particular grasp posture (e.g., a thumb-down grasp) by anticipating the intended action outcome (e.g., comfortably holding the cup upright with a thumb-up posture) based on the behavioral context (e.g., inverted cup in the shelf). So far, the DOF problem and the ABC framework have not been viewed together. Considering the combination of these two concepts (or aspects thereof), however, may benefit the understanding of the acquisition of motor planning skills for grip selection in young children.

Figure 1 depicts the three stages of proficiency in the OGT as a paradigmatic example of the acquisition of motor planning skills for grip selection, based on the adaptation of Hoffmann's ABC framework and Bernstein's stages of skill acquisition relative to the DOF problem. For an illustration, consider a child going to have a cup of milk. According to Hoffmann's ABC framework, the cup standing upside down on the shelf serves as the *situational condition*. The grasping action to be performed, namely to take the inverted cup from the shelf to have a cup of milk, is the *voluntary action*. The (body-internal) *effect anticipation* is the intended end-posture and the *real effect* is the re-afferent perception of the actual end-posture, which is attained when the manual rotation is completed. This defines the major components of the three-stage developmental model. Please note here, that dark gray or black shaded arrows and text in the graph marks these pathways to be present in the designated stage, the light gray shaded ones are not yet present in this stage of development.

**Figure 1:**
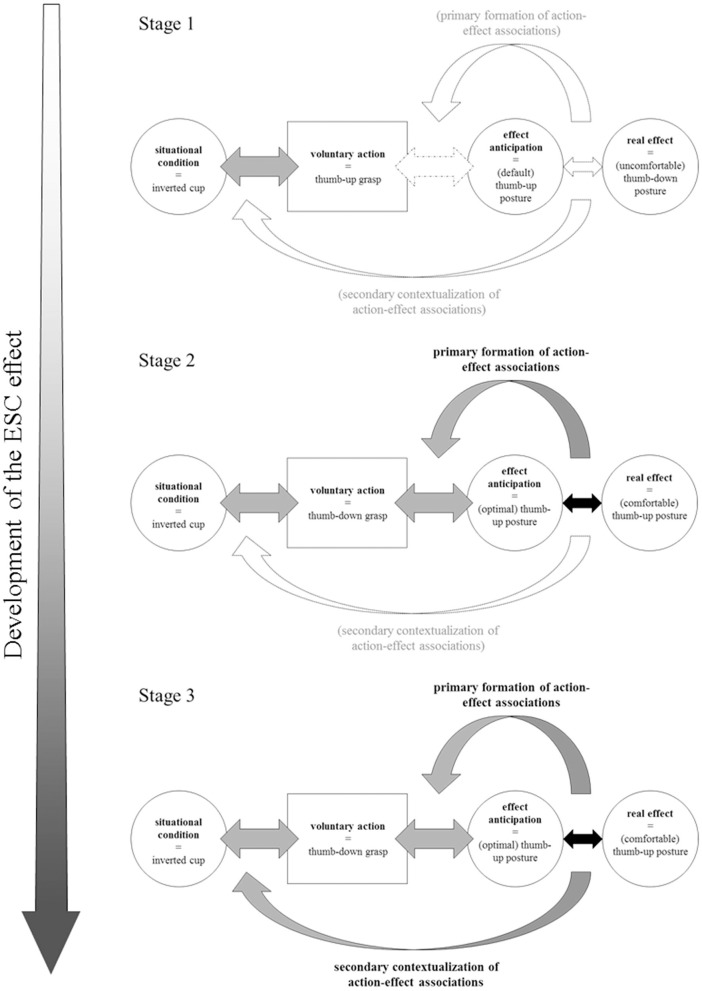
**Three-stage developmental model using an adaptation of the ABC theory by Hoffmann (2003) and Bernstein's (1967) stages of skill acquisition relative to the DOF problem, adjusted to account for children's performance in ESC tasks**. In Stage 1, children automatically select a default grasp (thumb-up grasp), as they are not able to anticipate other effects. As the real effect does not match the effect anticipation, no action-effect associations are formed and no contextualization to the situational condition takes place. In Stage 2, children are able to anticipate different action outcomes. Now, the real effect matches the effect anticipation and action-effect anticipations are formed (i.e., initial thumb-down grasp results in final thumb-up posture after rotation), but these are not yet contextualized to the situational condition. In Stage 3, children (and adults) are able to precisely anticipate desired action effects, based on strong action-effect associations. As the real effect reliably matches the effect anticipation, the action-effect association is now contextualized to the situational condition (i.e., inverted cup).

In Stage 1, children younger than (approximately) 3–4 years will most likely (habitually) select a default thumb-up grasp to reach for the cup, which can be inferred from the complete absence of the ESC effect in the large majority of children at this age (e.g., Weigelt and Schack, 2010; Jovanovic and Schwarzer, 2011). Such grasping behavior is an indication of first-order motor planning (i.e., selecting a grip relative to the immediate task demands), while second-order motor planning is lacking (i.e., selecting a grip according to what one wants to do with the object in the next step). Thus, they may not be able to anticipate an action outcome other than ending the movement in a thumb-up posture (as a default posture). This may be due to the “freezing” of additional DOF for better task control, as has been originally proposed by Bernstein (1967) and later on been confirmed in young children by Steenbergen et al. (1997). By “freezing” some additional DOFs for reaching and grasping, children select the most common grip posture for grasping the inverted cup in terms of first-order motor planning (i.e., the thumb-up posture) As a consequence, they will finish the manipulation in an uncomfortable thumb-down posture. Because experience with ESC tasks, gained through familiarization and/or trial repetition, does not seem to have a great influence on grasp selection in children of this age (Wunsch et al., 2013), the comparison of the real effects (i.e., uncomfortable thumb-down posture) with the effect anticipation (default thumb-up posture) in order to form action-effect associations may be incomplete, delayed, or may not take place at all. Therefore, neither the primary formation of action-effect associations nor the secondary contextualization of action-effect associations is realized in Stage 1.

In Stage 2, children between 5–10 years begin to “free” additional DOF as their motor actions become more variable, resulting in new task solutions (Bernstein, 1967). This is accompanied by eminent processes of motor reorganization (e.g., Meulenbroek and van Galen, 1988; Bard et al., 1990; Thibaut and Toussaint, 2010). Thus, from various experiences with different task solutions, they are now able to anticipate different action outcomes, such as a thumb-up posture (i.e., the optimal posture to finish the action). Here, the majority of children start to show the ESC effect for the first time. Accordingly, they are capable of second-order planning. Whenever this is the case, the real effect matches the effect anticipation and action-effect associations are formed (i.e., initial thumb-down grasp results in final thumb-up posture). This new action-effect association, however, is not yet contextualized to the situational condition (i.e., inverted cup). Therefore, the contingency between the most efficient grasp selection (initial thumb-down grasp) and the desired action effect (i.e., comfortable end-posture) is still weak and unstable. As a result, children will show large variability in their grasp selections during Stage 2 (Jongbloed-Pereboom et al., 2013; Wunsch et al., 2014).

In Stage 3, the optimal space of DOF is exploited (Bernstein, 1967) and grasping actions are flexibly selected to achieve intended goal-states. Children (typically) older than 10 years and adults are able to precisely anticipate desired (body-internal) action effects (i.e., to end comfortably with a thumb-up grasp; Stöckel et al., 2012), which is based on strong action-effect associations. Therefore, the real effect consistently matches the effect anticipation. Most importantly, these strong action-effect associations are contextualized to the situational condition (i.e., inverted cup in the shelf). The contextualization of different action-effect associations to different situational conditions allows for the flexible selection of grasping actions, which enables the child to choose the optimal grasp to reach comfortable end postures (Rosenbaum et al., 2001). Hence, anticipatory planning skills (as signified by the ESC effect) for grip selection during object manipulation are fully in place in Stage 3.

Some final considerations: Children may not perceive extreme joint angles as uncomfortable as adults do, because of their limber/more flexible limbs, as has been brought to attention by Rosenbaum et al. (2014). Therefore, it may be that the need for end-posture anticipation gets more important as children mature and their limbs become stiffer, postponing the presence of the ESC effect. Regarding the developmental trajectory of the present model, age ranges provided for the different stages are estimates or corridors based on previous findings (see Wunsch et al., 2013). However, the exact age at which children pass from one skill acquisition stage to another may depend on the specific ESC task used, as previous studies suggest that the age at which children reliably show ESC differs between tasks. For example, it seems that children show the ESC effect earlier in the OGT than in the bar-transport-task (Knudsen et al., 2012). There may also be a difference in the developmental trajectory between self-directed and other-directed actions (Claxton et al., 2009). More research is certainly needed with wider age ranges to assess anticipatory planning skills across different ESC tasks in order to make any predictions about the models' fit to different task versions. Also, the present considerations are limited to anticipatory planning skills for grip selection during object manipulation. There is evidence that children show advanced planning skills for complex motor actions at much earlier ages. For example, it was found that children as young as 12 months use complex alternative strategies to descend from heights, which do not afford their usual form of locomotion (Kretch and Adolph, 2013). Further, more skillful grip selection may rely on the child's cognitive development, a notion which is currently under a vivid debate (e.g., Van Swieten et al., 2010; Stöckel and Hughes, 2015). If the interdependency between cognitive and motor functions proves to be true, then this could help to understand the inter-individual differences in motor planning skills between children of the same age. Likewise, how long children remain in each acquisition stage and whether Stage 2 is just a (short) transition state is not clear and should be the focus of future research. At last, Stage 3 may be the final stage of motor planning skill acquisition, but the developmental pattern seems to reverse at the other end of the lifespan. This profound observation has been made in a most recent study, demonstrating the decline of the ESC effect at old ages (Wunsch et al., 2016). Considering both ends of the lifespan in future will complete the picture of the developmental pattern of acquiring anticipatory planning skills for grip selection in object manipulation.

## Author contributions

KW is the first author of this opinion article. She wrote the article by herself. MW is the second author of this article and participated in writing as well. The idea for the article was developed by both authors. Both authors made substantial contributions to the conception of the work and drafted and revised the paper critically for its intellectual content. Both authors approved the final version to be published. We agree to be accountable for all aspects of the work in ensuring that questions related to the accuracy or integrity of any part of the work are appropriately investigated and resolved.

## Funding

The article processing charge was funded by the German Research Foundation (DFG) and the Albert Ludwigs University Freiburg in the funding programme Open Access Publishing.

### Conflict of interest statement

The authors declare that the research was conducted in the absence of any commercial or financial relationships that could be construed as a potential conflict of interest.
